# New Jersey State Dental Society

**Published:** 1902-09

**Authors:** 


### NEW JERSEY STATE DENTAL SOCIETY.

The thirty-second annual meeting of the New Jersey State Dental Society convened at the Auditorium, Asbury Park, at half past ten o'clock, Wednesday, July 16, 1902, the President, Dr. Wm. L. Fish, of Newark, in the chair.

The attendance was unusually large. After the usual preliminaries, the Annual Address was read by the president. He first referred to matters of purely local interest and then invited attention to the large number and variety of dental exhibits, so well arranged in an adjoining room, perhaps the largest display ever seen at a State dental meeting. He spoke of its educational value as showing from year to year the advances made, and of its value to the members, giving them an opportunity to examine and become conversant with new instruments and appliances at their leisure, many of which may be helpful. If these were brought to their offices by an agent during business hours they would probably be curtly dismissed. He referred to the persistent and successful efforts to secure dental services for the army, and urged the Society to still support the efforts now being made to extend the same to the navy. Referring to the large increase in membership, he urged that every effort be made to bring out the young men, and to encourage them to take an active part in all professional work. The science of dentistry has become so broad that no one mind can compass all it embraces. It is therefore important that each should find for themselves that specialty for which they are best fitted, not only in practice, but also in society work, that they may be ready to take the place of those who year by year are called away. He spoke feelingly of the loss the profession had sustained during the past year by the death of many of its most prominent members.

Dr. Stockton, in discussing the address, spoke of the marvellous progress the world had made, and is now making. "It is only," he said, "by comparing the present with the past that this can be realized. We accept the changes this progress makes necessary as a matter of course, with but little thought of the enormous efforts which have brought them about." He referred to the years of study, the numberless experiments, and the immense labor before it was possible for Edison to light a room by touching a button, or to perfect his invention which enabled people a thousand miles apart to talk as freely as though face to face. Have we as a profession kept up with the procession? We do beautiful work, we have finely prepared gold, improved alloys, and numberless cements, but do we save more teeth than we did forty years ago? He answered in the negative, and suggested that the great need of to-day was a cement that could be readily adapted to the cavity, and that would remain there.

Dr. Meeker congratulated the Society upon its position as a leader among dental societies. Its earnestness, its progressiveness, and its success was due to promptly putting its new members to work. As soon as possible each new member is put upon some committee, it may be an unimportant one; it is, however, an entrance into society work. Those who show an ability and a disposition to work are advanced step by step, not as a compliment, but as a reward of merit. As a result, the affairs of the Society are managed by active, earnest, tried men; and the Society is an active society, constantly changing its workers, but never its methods, and always has some one ready to step in wherever a vacancy occurs.

After the transaction of routine business the meeting adjourned until evening, the afternoon session being omitted to allow the members to become acquainted with their surroundings. Many spent the afternoon examining the exhibits in the large hall adjoining the meeting-room.

The first paper at the evening session, by Dr. J. Lenox Curtis, of New York City, was entitled "Electric Ozonation in the Treatment of Neuralgia." This was upon a somewhat new application of electricity, modified so as to utilize an interrupted current of very high voltage and low amperage for the detection and cure of diseased conditions. The apparatus used was very similar to that by which X-rays are produced and Geisler tubes operated. When in operation ozone is freely generated, making it an effective sterilizer, and as such it may be used to purify the atmosphere of a room, or by proper manipulation of suitable adjuncts to have a local antiseptic effect. It is said to relieve certain forms of neuralgia by removing their cause, to be useful in many diseased conditions by bringing about the destruction of broken-down tissue and stimulating repair, and to be especially valuable in promptly detecting actual or threatened morbid conditions.

This was followed by a paper entitled "Ethnographic Odontography," written by Dr. Alton Howard Thompson, of Topeka, Kan. In the absence of the writer, it was read by Dr. C. S. Stockton. The paper was a purely technical one, and, however valuable it may be, was not suitable for a State dental meeting. It is a mistake to present such a paper read by any one but the author. Dr. Stockton probably read it as well and as effectively as any one but the author could have done; no one can do justice to a subject with which he is not familiar. Such matters should be presented in the journals direct, where they may be read carefully and at leisure. However important the subject may be, or however well presented at a dental meeting, it is one experts only can discuss; unless such papers are very short and terse, and interestingly written, they are only time and patience wasters.

This ended the evening session.

The morning and evening sessions of Thursday were mainly occupied with the reading and discussion of a paper by Dr. R. Ottolengui, of New York City, entitled "Should Children's Teeth be filled with Gold?"

The doctor's position, as understood by the writer, was that, so far as recurring decay was concerned, there was nothing in the teeth themselves to counterindicate the use of gold for filling carious cavities of children's teeth. With proper attention to cavity preparation, especially with a view to protect its margins, gold may be inserted with full confidence that it will prove an effective and lasting operation. He fully appreciated the limits imposed by the little patient's ability to bear the necessary manipulation, and the importance of properly protecting immature portions of the tooth; and he dealt with the question of its expediency in those cases only where it could be properly placed. In such cases, he contended, it made the best possible repair. He supported his position by reading notes endorsing his conclusions from well-known dental histologists. The discussion was quite animated, but the matter has so often been gone over that very little, if anything, new and directly pertinent to the question was brought out. The real point of the essay was missed, and argument had upon matters which the essayist had expressly excluded. He freely admitted that there were many cases where, on other accounts than incompatibility of gold and children's teeth, gold was not the best.

The clinics on Thursday afternoon, I was impressed, were not as instructive as usual. They were scattered, the clinicians were difficult to find, and most of them were in positions so confined that but few were able to see what was going on. To make dental clinics what they should be at a meeting like this is an exceedingly difficult problem to solve; especially so when, as in this case, thirty-four had to be provided for. This difficulty is increased when, as usually happens, some fail to appear.

The exhibits were well arranged, and formed a very attractive feature of the meeting. I noticed but few devices that could be considered really new. Gideon Sibley, of Philadelphia, exhibited his latest improved chair. It has a range of from sixteen to thirty-seven and a half inches in height, and a large number of movements, all controlled by cams, making it easy to operate and very convenient.

The Buffalo Dental Manufacturing Company had a set of bolts for vulcanizing flasks, each provided with a spring, making a very simple arrangement for closing the flask by spring pressure without adding to the usual equipment. They are sold at the same price as those in ordinary use, and may be used on nearly all makes of flasks on the market. The advantage of spring pressure in closing flasks, saving fracture of casts and teeth, and saving time, is very great, especially so when applied at three points. They are well worth a trial. They also make a variety of brass nozzles to screw on gas-fixtures for conveniently attaching rubber hose, some straight, others more or less curved, to prevent the hose "kinking."

I noted with much pleasure an exhibit by P. Blakiston's Son & Co., and another by Lea Brothers, both of Philadelphia, of a choice selection of books a dentist should have and read. Both exhibits were in charge of gentlemen well posted as to the relative merits of the works shown, and well able and willing to give any information desired. Few dentists, especially those living at a distance from the larger cities, are posted, or have opportunity to conveniently make themselves familiar with the latest or the best professional literature. It was a pleasure to see so many avail themselves of the opportunity thus offered, and to listen to the suggestions of those in charge, who played the rôle of exhibitors rather than salesmen, and to further see the memorandum book, in which orders were entered, so often used. If there is any one thing the profession needs more than another, it is more readers,—larger libraries in dental offices and a better acquaintance with the current professional literature of the day.

It required a full half-day to do justice to the exhibits, and was a full half-day well and profitably spent.

The last day was occupied with society business. The following officers were elected: President, Frank L. Hindle, of New Brunswick; Vice-President, Herbert S. Sutphen, of Newark; Secretary, Charles A. Meeker, of Newark; Treasurer, Henry A. Hull, of New Brunswick.

